# Relationship between Body Mass Index and *T*-Scores of Bone Mineral Density in the Hip and Spine Regions among Older Adults with Diabetes: A Retrospective Review

**DOI:** 10.1155/2019/9827403

**Published:** 2019-04-22

**Authors:** Abdulaziz F. Hariri, Mohammad N. Almatrafi, Aws B. Zamka, Abdullah S. Babaker, Tariq M. Fallatah, Omar H. Althouwaibi, Amre S. Hamdi

**Affiliations:** ^1^Faculty of Medicine, King Abdulaziz University, Jeddah, Saudi Arabia; ^2^Faculty of Medicine, University of Jeddah, Jeddah, Saudi Arabia; ^3^Consultant and Assistant Professor of Orthopedic Surgery, Faculty of Medicine, King Abdulaziz University, Jeddah, Saudi Arabia

## Abstract

Diabetes mellitus (DM) cases are increasing worldwide, especially in Saudi Arabia. Previous studies suggested a positive relationship between body mass index (BMI) and bone mineral density (BMD) levels. Generally, patients with low BMI (<18.5 kg/m^2^) have reduced BMD levels and, thus, low *T*-scores; hence, they are categorized as osteopenic or osteoporotic. In this study, we aimed to determine whether a relationship between BMI and BMD *T*-scores in the hip and spine regions of patients with diabetes exists. This retrospective record review investigated older adult patients with diabetes in King Abdulaziz University Hospital (*n*=198; age 50–90 years) who underwent BMD scan between January 1, 2016, and June 25, 2018, regardless of their sex but limited to type 2 DM. The height and weight of all subjects were recorded, and BMI was calculated and categorized. We used SPSS version 21 for data analysis; measures of central tendencies, Pearson's correlations, chi-square tests, and independent *t*-tests were employed. We found positive relationships between BMI and BMD *T*-scores in the hip and spine regions (right femoral neck: *R*=+0.214, *P* ≤ 0.002; total right hip: *R*=+0.912, *P* ≤ 0.001; left femoral neck: *R*=+0.939, *P* ≤ 0.001; total left hip: *R*=+0.885, *P* ≤ 0.001; and total lumbar region: *R*=+0.607, *P* ≤ 0.001). Low BMI (<18.5 kg/m^2^) could be a risk factor for osteoporosis, whereas normal/high BMI could be protective against osteoporosis among adults with diabetes.

## 1. Introduction

Diabetes mellitus (DM) cases are increasing worldwide. DM results from dysfunction in glucose metabolism and has different classifications depending on the pathophysiological cause: type 1 DM (T1DM) is caused by insulin deprivation, and type 2 DM (T2DM) is caused by insulin desensitization accompanied by insufficient insulin production [1]. A retrospective study in the United States of America (USA) published in 2015 reported an estimated DM prevalence of 12–14% between 2011 and 2012. In Saudi Arabia, a community-based research showed a total prevalence of 23.7%. Another recent local study showed that T2DM alone had a prevalence of 17.7% in men and 16.4% in women [2–5].

A comparative study in the USA concluded that a directly proportional relationship between body mass index (BMI), which is calculated as weight in kilograms divided by height in meters squared (kg/m^2^) [6], and DM prevalence exists [7]. A recent study in older adults that was conducted to explore the relationship between BMI and T2DM showed that a high BMI is considered a risk factor for T2DM complications. A BMI >25 kg/m^2^ is considered a predisposing factor for T2DM, and those with a BMI >30 kg/m^2^ have a 100% risk of developing T2DM compared with those with normal BMI [8].

Moreover, several studies demonstrated the relationship between low BMI, low bone mineral density (BMD) levels, and the risk of osteoporotic fractures [9–11], and some studies found that increased BMI is associated with elevated BMD levels and a reduced risk of fractures due to osteoporosis [12–14].

The major complications of DM include heart attack, stroke, kidney failure, blindness, and lower limb amputation [15, 16]. T1DM patients have reduced BMD levels and a high risk of fractures, which are attributable to the reduction of bone formation markers and increased bone resorption markers. In T2DM patients, despite their relatively higher BMD levels, their fracture risk is similar to that in T1DM patients [17–21].

Furthermore, osteoporosis is one of the most prevalent diseases in the older population [22] and is caused by decreased bone quality and BMD [23]. BMD levels may reflect the skeletal condition of the body and could predict the probability of osteoporotic fractures [24, 25]. The World Health Organization (WHO) provided the following classification based on *T*-scores, which represent the number of standard deviations below or above the average BMD: normal (>−1.0), osteopenic (−1.1 to −2.5), and osteoporotic (≤−2.5) [26]. The prevalence of osteoporosis in Saudi Arabia in both genders aged >55 years is >30% [27, 28]. Osteoporosis along with osteopenia is responsible for fragility fractures, which contribute to the increasing morbidity and mortality rates. A previous study published in 2015 showed that the proximal femoral fracture incidence is 5.89 per 1000 individuals, and the estimated lifetime spending for fragility femoral fractures was 9.34 billion USD [29–31]. Moreover, 17 billion USD was the total healthcare cost of two million cases of fractures in the USA alone, and such cost is calculated to increase to 25 billion USD in 2025 because of an estimated 50% increase in fracture incidence [32].

Further studies are needed to explore the relationship between BMI and BMD levels, especially among older diabetic patients. To our knowledge, no relevant studies were conducted in Saudi Arabia. Thus, in this study, we aimed to determine the relationship between BMI and the BMD *T*-scores in the hip and spine regions of older adult diabetic patients in King Abdulaziz University Hospital and to compare the BMD levels and the corresponding *T*-scores between the regions.

## 2. Methods

### 2.1. Ethical Approval

Ethical clearance was obtained from the Institutional Review Board (IRB) of King Abdulaziz University Hospital.

#### 2.1.1. Study Design and Population

This is a retrospective record review of older adult diabetic patients (aged 50–90 years), regardless of ethnicity, nationality, sex, or type of DM, who underwent bone mineral densitometric scans via dual-energy X-ray absorptiometry (DEXA) between January 1, 2016, and June 25, 2018; those with comorbidities such as hyperthyroidism, rheumatoid arthritis, end-stage renal disease, chronic renal disease, diabetic nephropathy, and Addison's disease; those who had undergone vertebrae fixation; and those who were receiving glucocorticoids, antiepileptics, chemotherapy, androgen antagonists, aromatase inhibitors, or anticoagulants were excluded.

#### 2.1.2. Data Collection and Availability

Our convenience sample was composed of 550 subjects. This study was conducted at the King Abdulaziz University Hospital in Jeddah City, Saudi Arabia. We used a data collection sheet for our primary data, which contained the following parts: (1) demographic data: age, sex, ethnicity, weight, height, BMI values, and nationality; (2) type of DM: type 1 or type 2; and (3) BMD scan data: BMD levels and their corresponding *T*-scores at the right hip (femoral neck and total hip), left hip (femoral neck and total hip), and lumbar vertebrae (L1-L2 and total lumbar region).

We obtained and filtered the data using the hospital system according to our inclusion and exclusion criteria. Data were kept confidential, sealed with a passcode, and are available from the corresponding author upon request.

#### 2.1.3. Data Entry and Analysis

We used Microsoft Excel® for data entry and the Statistical Package for the Social Sciences version 21 (SPSS Inc., Chicago, IL, USA) for data analysis. Chi-square and independent *t*-tests, measures of central tendencies, and Pearson's correlations were employed, and all statistical test results were considered significant if the *P* value was less than 0.05; regarding the Pearson's coefficient (*r*) value, if the result was 0, it is considered as no relationship, weak strength relationship if less than 0.2, moderate strength relationship if between 0.2 and 0.4, and strong strength relationship if more than 0.4. The interpretation of the signs positive (+) and negative (−) depends on the two variables' signs that are being studied; as if both were positive/negative, it would indicate a direct proportional relationship, but if either one was positive or negative, the other was the opposite, and it would indicate an inverse relationship.

#### 2.1.4. DEXA and *T*-Scores

DEXA scan, which is a standard way of assessing BMD, could provide data on fracture risk and *T*-scores. According to the WHO, *T*-scores represent the number of standard deviations below or above the average BMD. Based on the *T*-scores, patients are classified as follows: normal (>−1.0), osteopenic (−1.0 to −2.5), and osteoporotic (≤−2.5) [26, 33]. BMD was expressed in g/cm^2^.

#### 2.1.5. BMI Categories

Weight, height, and BMI were obtained on site and before DEXA scans. The patients were classified according to the WHO classification [34]: underweight (<18.5 kg/m^2^), normal weight (18.5–24.9 kg/m^2^), overweight (25.0–29.9 kg/m^2^), obesity class I (30.0–34.99 kg/m^2^), obesity class II (35.0–39.99 kg/m^2^), and obesity class III (>40.0 kg/m^2^).

## 3. Results

**Figure 1:**
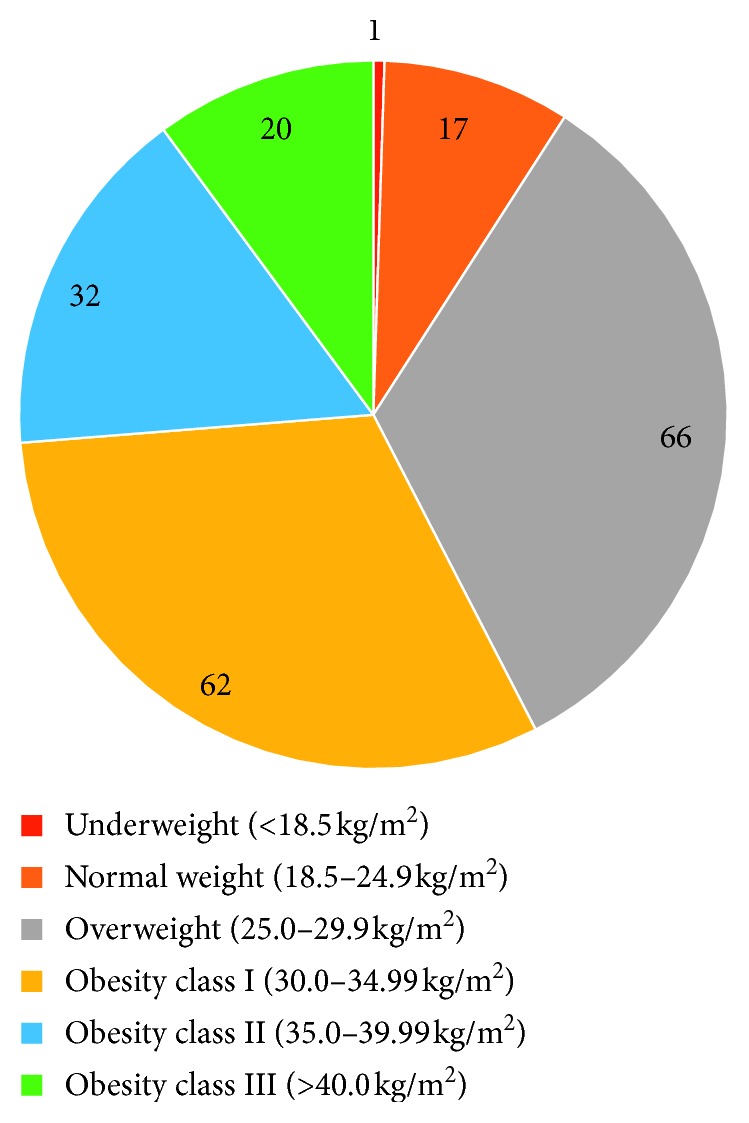
BMI categories of our sample.

Of the 550 files we have retrieved and after applying the inclusion and exclusion criteria, 198 patients were included in the analysis (women, 177 (88.50%); men, 21 (11.50%)). The demographic data are shown in Table 1 and Figure 1.

### 3.1. Relationship between BMI and BMD *T*-Scores in the Hip and Spine Regions

**Figure 2:**
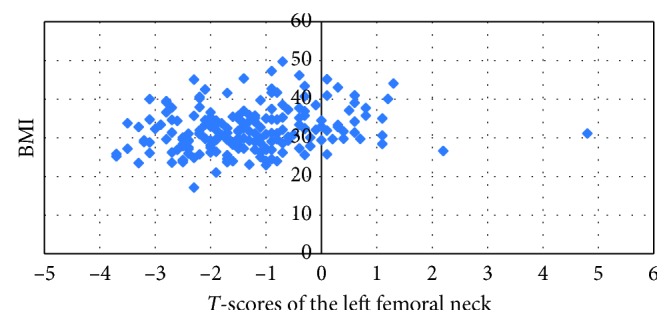
Correlation between BMI values and *T*-scores of the left femoral neck.

All relationships were strong positive significant relationships between BMI and BMD *T*-scores in the right total hip (*R*=+0.912, *P* ≤ 0.001), the left femoral neck (*R*=+0.939, *P* ≤ 0.001), total left hip (*R*=+0.885, *P* ≤ 0.001), and lumbar 1 (*R*=+0.590, *P* ≤ 0.001) and 2 vertebrae (*R*=+0.587, *P* ≤ 0.001); the total lumbar region (*R*=+0.607, *P* ≤ 0.001) was noted based on the Pearson's correlation expect for the right femoral neck which had a moderate positive relationship (*R*=+0.214, *P* ≤ 0.002). Correlations between BMI and BMD *T*-scores are summarized in Table 2 and Figure 2.

### 3.2. Normative Comparison of BMI and BMD *T*-Scores in the Hip and Spine Regions according to Sex

The lowest means of BMD in both sexes were found in the left femoral neck (men, 0.766 ± 0.1428; women, 0.702 ± 0.1355 (both *P* ≤ 0.042). The highest means of both BMD and *T*-scores in men were in the total lumbar region (1.047 ± 0.1794, *P* ≤ 0.001 and −0.367 ± 1.6788, *P* ≤ 0.001, respectively). The lowest mean of *T*-scores in women was in the total lumbar region (−1.460 ± 1.3160, *P* ≤ 0.001) (correlations between BMD levels, *T*-scores, and sex are summarized in Table 3).

## 4. Discussion

Our study's population, sample size, and demographic characteristics differ from other similar studies. For example, a previous study in China was conducted to identify the relationship between BMI and BMD; the study had two groups of postmenopausal women only (T2DM group and control group) [35]. Nevertheless, our sample is similar due to the great disparity in relation to the female to male ratio; that is, probably due to being a woman itself regardless of age, it is more associated with low BMD compared with men. Thus, DEXA scan for women is a priority according to the US Preventive Services Task Force, especially after menopause [36].

All correlations between BMI and BMD *T*-scores in the hip and spine regions were significantly directly proportional although the correlation strength varied between the strong and moderate, which means that an increase in BMI could result in increased BMD *T*-scores, and vice versa. Both the right femoral neck and total right hip had weak correlations. The correlations in all relationships were strong positive significant relationships (the right total hip, the left femoral neck, total left hip, lumbar 1 and 2 vertebrae, and total lumbar region in comparison to the right femoral neck which showed moderate positive relationship). Previous studies concluded that when BMI increases, BMD levels will also increase, which further supports our findings [37, 38]. Moreover, other studies explained that such relationship exists because heavy body weight could result in bone remodeling to compensate for the heavy mechanical load [39, 40]. Another study suggested that an increased BMI could subsequently increase the levels of leptin, which contributes to the relationship by promoting osteoblast production and functions [41–44]. Other studies showed that early postmenopausal women with a low BMI have low BMD compared with women with a higher BMI, further supporting the positive relationship between the two variables [13, 45].

Majority of our subjects are classified as overweight and obesity class I and II (mean BMI = 31.96 kg/m^2^), possibly because some of the patients are on insulin therapy (insulin is an anabolic hormone that could increase weight) and because of the sedentary lifestyle and unhealthy dietary choices of patients.

Comparing between both sexes' means of *T*-scores in the hip and spine regions, the lowest mean of *T*-scores in men was in the left femoral neck, and the lowest and highest means of *T*-scores in women and men, respectively, were in the total lumbar region. The highest mean of *T*-scores in women was in the total right hip region. According to the WHO, an increase in *T*-score indicates a worsening bone condition (i.e., the more negative the score is, the worse the category is) [26].

Recent similar studies published in 2012, 2016, and 2018 in the USA, China, and India, respectively, compared the means of BMD and *T*-scores, but their studied population differed. For instance, the study in the USA involved only women who were subcategorized according to their insulin dependency, and the study in China and that in India included both sexes, but neither of them was diabetic; moreover, the age range in the study in India was different from that in our study.

We noticed that the means of BMD *T*-scores in the regions of interest in our study were worse than those in previous studies [46–48], probably because all our subjects are diagnosed with type 2 diabetes, and DM, as demonstrated in previous studies, is a contributing factor to such difference because of its effect at the cellular level. Prolonged hyperglycemia is found to affect both the function and quantity of osteoblasts by disrupting the response to vitamin D and stimulating glycation of multiple proteins. Glycation produces end products that could accumulate and be embedded into the bony matrix and consequently damage the bones [16, 49–54]. Moreover, another possible reason could be the duration of DM and menopause. However, in our study, relevant data were missing in the records, thereby raising the probability of confounders. In addition, the differences in nationalities, ethnicities, and genetic makeup may also play a role. Thus, we could hypothesize that, generally, Saudis and/or Arabs may have lower BMD levels than Americans, Chinese, or Indians; however, this should be further explored in future identical comparative study.

This study has some unavoidable limitations, especially with its retrospective nature. First, the number of female subjects in this study is significantly greater than that of male subjects, which could be because the female sex is considered a risk factor for low BMD at any age and especially after menopause [36]. Second, this study focused on T2DM as a whole, and we did exclude those who were diagnosed with T1DM because of a significant numerical disparity between T1DM and T2DM patients in the data we retrieved and to assess and focus more on T2DM in specific. Third, we could not determine how long the subjects have been diagnosed as having DM as well as the duration of menopause. Fourth, not all lumbar region vertebrae were scanned; we found that scan data for L3 and L4 are missing in some patients. This could be because not all patients received the same treatment and a portion of patients had missing vertebrae congenitally. Hence, we excluded L3 and L4 to avoid missing data bias.

## 5. Conclusions

Diabetic patients with high BMI possibly have a lower risk of osteoporosis than those with low BMI. All patients with diabetes should be encouraged and educated about controlling their diabetes and maintaining normal BMI or increasing BMI for those with low BMD by having well-balanced and healthy diets to prevent the risk of fragility fractures and osteoporosis.

Finally, a local BMD screening program for older adults with DM is highly encouraged for early detection of osteoporotic risk and to prevent further complications. Future studies with a larger sample size are warranted to further explore the relationship between BMI and BMD *T*-scores of people with diabetes and the pathophysiological mechanisms.

## Figures and Tables

**Table 1:** Demographic characteristics of the patients with type 2 diabetes mellitus.

Categories	T2DM subjects (*n*=198)
Age (years)	63.64 ± 8.249
Height (cm)	154.42 ± 8.04
Weight (kg)	76.14 ± 14.23
BMI (kg/m^2^)	31.96 ± 5.57
BMI categories	
Underweight (<18.5 kg/m^2^)	1
Normal weight (18.5–24.9 kg/m^2^)	17
Overweight (25.0–29.9 kg/m^2^)	66
Obesity class I (30.0–34.99 kg/m^2^)	62
Obesity class II (35.0–39.99 kg/m^2^)	32
Obesity class III (>40.0 kg/m^2^)	20
Nationality	
Saudis	100
Non-Saudis	98
Ethnicity	
Arabs	182
Non-Arabs	16
Sex	
Male	21
Female	177

T2DM: type 2 diabetes mellitus. Values are presented as mean ± SD.

**Table 2:** Relationship between BMI and BMD *T*-scores in the hip and spine regions.

*T*-score regions	*R* values	*P* values
Right femoral neck	+0.214	≤0.002
Total right hip region	+0.912	≤0.001
Left femoral neck	+0.939	≤0.001
Left total hip	+0.885	≤0.001
Lumbar 1 vertebra	+0.590	≤0.001
Lumbar 2 vertebra	+0.587	≤0.001
Total lumbar region	+0.607	≤0.001

*R*: Pearson's correlation coefficients; *P*: partial correlation coefficients.

**Table 3:** Pearson's correlations between BMD levels, *T*-scores, and sex.

Bone parameters	Men (*n*=21)	Women (*n*=177)	*P* values
Right femoral neck			
BMD (g/cm^2^)	0.784 ± 0.1323	0.7127 ± 0.1422	≤0.028
*T*-score	−1.029 ± 0.1035	−1.270 ± 1.2662	≤0.402
Total right hip region			
BMD (g/cm^2^)	0.921 ± 0.1407	0.8263 ± 0.1524	≤0.007
*T*-score	−0.714 ± 0.9759	−0.998 ± 1.1996	≤0.298
Left femoral neck			
BMD (g/cm^2^)	0.766 ± 0.1428	0.702 ± 0.1355	≤0.042
*T*-score	−1.167 ± 1.0947	−1.350 ± 1.2129	≤0.510
Total left hip region			
BMD (g/cm^2^)	0.911 ± 0.1511	0.819 ± 0.1443	≤0.007
*T*-score	−0.762 ± 1.0447	−1.048 ± 1.1505	≤0.278
Lumbar 1 vertebra			
BMD (g/cm^2^)	0.983 ± 0.1717	0.861 ± 0.1465	≤0.001
*T*-score	−0.668 ± 1.5981	−1.224 ± 1.3278	≤0.078
Lumbar 2 vertebra			
BMD (g/cm^2^)	1.037 ± 0.2041	0.8912 ± 0.1539	≤0.001
*T*-score	−0.481 ± 1.886	−1.302 ± 1.4101	≤0.016
Total lumbar region			
BMD (g/cm^2^)	1.047 ± 0.1794	0.8911 ± 0.1482	≤0.001
*T*-score	−0.367 ± 1.6788	−1.460 ± 1.3160	≤0.001

Values are presented as mean ± SD.
